# A tool to balance benefit and harm when deciding about adjuvant therapy

**DOI:** 10.1038/sj.bjc.6604962

**Published:** 2009-03-17

**Authors:** A M Knops, A Goossens, M P M Burger, L J A Stalpers, D T Ubbink

**Affiliations:** 1 Department of Quality Assurance and Process Innovation, Academic Medical Center at the University of Amsterdam, The Netherlands; 2 Department of Gynaecology, Academic Medical Center at the University of Amsterdam, The Netherlands; 3 Department of Radiotherapy, Academic Medical Center at the University of Amsterdam, The Netherlands

**Keywords:** absolute risk increase, absolute risk reduction, adjuvant radiotherapy, decision making, endometrial carcinoma

## Abstract

Adjuvant therapy aims to prevent outgrowth of residual disease but can induce serious side effects. Weighing conflicting treatment effects and communicating this information with patients is not elementary. This study presents a scheme balancing benefit and harm of adjuvant therapy *vs* no adjuvant therapy. It is illustrated by the available evidence on adjuvant pelvic external beam radiotherapy (RT) for intermediate-risk stage I endometrial carcinoma patients. The scheme comprises five outcome possibilities of adjuvant therapy: patients who benefit from adjuvant therapy (some at the cost of complications) *vs* those who neither benefit nor contract complications, those who do not benefit but contract severe complications, or those who die. Using absolute risk differences, a fictive cohort of 1000 patients receiving adjuvant RT is categorised. Three large randomised clinical trials were included. Recurrences will be prevented by adjuvant RT in 60 patients, a majority of 908 patients will neither benefit nor suffer severe radiation-induced harm but 28 patients will suffer severe complications due to adjuvant RT and an expected four patients will die. This scheme readily summarises the different possible treatment outcomes and can be of practical value for clinicians and patients in decision making about adjuvant therapies.

Adjuvant therapy aims to prevent outgrowth of residual disease after surgical removal of a malignancy to improve disease-specific and overall survival. However, adjuvant therapy can induce serious side effects as well. Therefore, the benefit of adjuvant therapy in terms of a reduced risk of recurrence needs to be weighed carefully against the risk of therapy-induced harm, before a decision is made.

Particularly when the effect of available treatment options is ambiguous or equal, patient preferences can play a crucial role in decision making. In a recent study by Pieterse *et al* (2007), the deciding benefit to choose for neoadjuvant radiotherapy (RT) and the importance of different treatment outcomes were found to vary widely among rectal cancer patients as well as between oncologists and patients. Moreover, oncology patients were found to be more than willing to participate in decision making after being thoroughly informed (van Tol-Geerdink *et al*, 2006). Clinicians should therefore adequately inform their patients about the risks and benefits of adjuvant therapy, and elicit patient preferences regarding treatment outcomes.

It is not elementary to weigh conflicting treatment effects and to communicate this information with patients. Parameters such as number needed to treat (NNT) and number needed to harm (NNH) have been proposed to give the clinician some direction of the benefit and harm of adjuvant therapies. However, they often over-simplify the consequences of a treatment (Nuovo *et al*, 2002; Massel, 2003). For example, NNT does not differentiate between patients in whom a recurrence is prevented without suffering side effects and patients who are spared from a recurrence but who do suffer serious side effects. Thus, these rather intangible numbers cannot express how many patients will benefit or suffer from the adjuvant therapy.

In intermediate-risk stage I endometrial carcinoma, for instance, adjuvant pelvic external beam RT is presently the acknowledged treatment to reduce the risk of outgrowth of residual disease in the pelvis (http://www.guideline.gov/summary/summary.aspx?doc_id=9203&nbr=004957&string=endometrial+AND+cancer). Nevertheless, a great international controversy exists (Burger, 2001; Johnson and Cornes, 2007). In a recent meta-analysis, the benefits of adjuvant RT were reviewed. In the subgroup of intermediate-risk patients, a statistically significant reduction of local recurrences was observed when adjuvant RT was applied compared with no adjuvant therapy following surgery (NNT 16.7 women; 95% confidence interval 12.5–25.0). However, this advantage did not result in a better overall or endometrial carcinoma-related survival for these patients (Kong *et al*, 2007). No clear recommendations for clinical practice were given based on these results.

A plain scheme presenting (the risk of) all possible outcomes of adjuvant therapy could provide more insights for clinicians as well as for patients. Such a tool has been designed earlier with respect to neoadjuvant RT for resectable rectal carcinomas (Bakx *et al*, 2006). Yet, this has not been recognised as a useful tool to facilitate clinician–patient communication when vacillating about the decision for (neo)adjuvant cancer therapy.

The aim of this study is to present a scheme balancing the benefit and harm of adjuvant therapy *vs* no adjuvant therapy, illustrated by the available evidence on adjuvant pelvic external beam RT for intermediate-risk stage I endometrial carcinoma patients.

## Materials and methods

The scheme comprises five outcome possibilities of adjuvant therapy, ranging from patients experiencing full benefit without any harm to patients experiencing full harm, that is death from adjuvant therapy (Table 1). Presenting these different outcome possibilities in natural frequencies is not yet very common, but helpful (Hoffrage *et al*, 2000). Therefore, evidence on the beneficial and harmful effects of adjuvant RT was needed to enter data into the scheme.

All available relevant evidence was searched in PubMed, and studies were included if they comprised (1) randomised clinical trials (RCTs), (2) comparing adjuvant pelvic external beam RT following surgery *vs* surgery alone and (3) in women with stage 1 intermediate-risk endometrial carcinoma. Local recurrences and RT-induced harm had to be one of the clinical end-points measured.

The extent of benefit and harm in the included studies was determined by means of the absolute risk reduction (ARR) and absolute risk increase (ARI) (McAlister *et al*, 2000; Straus, 2002). Treatment benefit can be expressed as an ARR. This expresses the absolute additional beneficial effect of adjuvant therapy over no adjuvant treatment and is equal to the difference between control event rate and experimental event rate found in RCTs (ARR=event rate control group−event rate intervention group). The ARR can also be calculated as the reciprocal of NNT, which is commonly used to express the number of patients who needs to be treated to gain one additional beneficial outcome. For instance, an NNT of 5 corresponds to an ARR of 1/5=0.2.

Although survival is considered to be the ultimate primary outcome in oncology trials, the available RCTs considering adjuvant RT in stage I intermediate-risk endometrial cancer patients have shown neither overall survival benefit nor endometrial-carcinoma-related survival advantage. Hence, in this analysis, we chose local recurrence rate (ARR_local recurrence_) as the primary outcome parameter. Preventing local recurrences is relevant if it increases the rate of cancers cured and if it avoids the women's mental and physical morbidity of additional diagnosis and treatment (Creutzberg *et al*, 2000).

The ARI is a measure of treatment-induced harm. It is defined by the absolute difference in complication rates between two treatment modalities (ARI=complication rate intervention group−complication rate control group). The ARI can also be calculated as the reciprocal of NNH, which is used to express the number of patients who has to undergo treatment to induce one additional harmful event. In this analysis, the ARI was the difference between the complication rates of women with and without adjuvant RT. Complication rates involved morbidity and mortality, resulting in ARI_morbidity_ and ARI_mortality_.

Although mortality is a clear-cut end-point, morbidity may be subject to discussion regarding the level of severity. In this analysis, only severe grade 3 complications, according to the French–Italian glossary, were taken into account (Chassagne *et al*, 1993), because the severity of these complications could cause women to reconsider their decision about having adjuvant RT. Grade 3 complications are life-threatening *per se* or due to their treatment required. They also comprise any permanent and severe tissue or organ damage (e.g., stenosis, bleeding, or fistulas of the intestines or of the urinary tract). Grade 4 complications are deaths due to a complication of the treatment of cancer, comprising ARI_mortality_.

From the included studies, the reported number of study participants in the RT and control groups, the number of local recurrences, severe complications and deaths due to adjuvant RT were extracted (Table 2). These were combined into an overall ARR, overall ARI for morbidity and overall ARI for mortality. A 95% CI was determined to provide an estimate of the precision of the ARR and ARIs.

Subsequently, patients receiving adjuvant RT were divided into five outcome groups based on the benefit and harm the therapy may induce. As using absolute numbers and stating the reference class is likely to improve the understanding of risks and benefits (Gigerenzer and Edwards, 2003), this is illustrated in a fictive cohort of 1000 women receiving adjuvant RT. It is worth noting that the ‘benefit’ in this analysis is about the difference in local recurrence rates between the two treatment modalities, whereas ‘harm’ in this scheme concerns the difference in complication rates. Table 1 gives the equations to calculate each of the five groups (Schulzer and Mancini, 1996; Mancini and Schulzer, 1999).

## Results

Six RCTs, studying adjuvant pelvic external beam RT *vs* no adjuvant therapy in early-stage endometrial carcinoma, were identified (Aalders *et al*, 1980; Weigensberg, 1984; Creutzberg *et al*, 2000; Soderini *et al*, 2003; Keys *et al*, 2004; the ASTEC/EN.5 writing committee 2008, 2009). One RCT was excluded because the control group underwent another adjuvant intervention instead of no intervention (Aalders *et al*, 1980). The clinical trial of Weigensberg (1984) focussed on preoperative RT (Weigensberg, 1984). The study of Soderini *et al* (2003) (published as an abstract only) did not report radiation-induced harm, and was therefore also excluded from analysis (Soderini *et al*, 2003).

Three large RCTs remained: the Post Operative Radiation Therapy in Endometrial Carcinoma-1 study from Creutzberg (PORTEC-1), the Gynecologic Oncology Group 99 study from Keys (GOG 99) and A Study in the Treatment of Endometrial Cancer/EN.5 study from the ASTEC/EN.5 writing committee 2008 (ASTEC/EN.5) (Creutzberg *et al*, 2000; Keys *et al*, 2004; the ASTEC/EN.5 writing committee 2008, 2009). They comprised 2011 women, and relevant data on benefit and harm in the intervention and control groups could be extracted (Table 2).

All studies were of a randomised design, compared the same interventions (adjuvant pelvic external beam RT *vs* no adjuvant therapy) and reported the required outcome measures (local recurrence rate and treatment-induced harm) on similar patients (intermediate-risk stage I endometrial cancer patients). In the ASTEC/EN.5 trial, brachytherapy was also allowed if the centre's policy was to offer it to all International Federation of Gynecology and Obstetrics (FIGO) stage I or IIA women irrespective of RT allocation. Similar proportions of women in the RT group as well as the control group received brachytherapy (52 and 51%, respectively). Providing brachytherapy might lead to an underestimation of the beneficial effect of adjuvant external RT and an overestimation of the risk of complications of adjuvant RT for stage I intermediate-risk endometrial cancer patients. However, sensitivity analyses showed that the numbers in the model hardly changed when only the PORTEC-1 and GOG 99 trials were taken into account (ARR 0.09, ARI_morbidity_ 0.04 and ARI_mortality_ 0.005). All in all, we decided that the data of these three studies could be used to calculate an overall ARR and ARI to complete our scheme.

**Figure 1:**
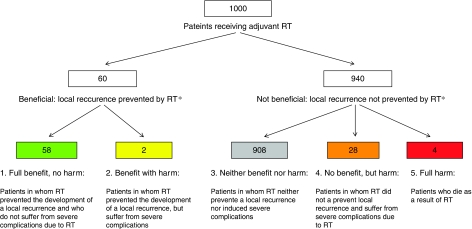
Benefit and harm for a fictive cohort of 1000 irradiated patients in whom adjuvant RT is compared with no adjuvant treatment. ^*^This figure displays the differences between adjuvant RT and no adjuvant therapy rather than absolute frequencies, that is the additional benefit in terms of local recurrences as prevented by adjuvant RT and the additional harm in terms of severe complications and deaths as induced by adjuvant RT.

Subsequently, the overall ARR and ARIs were combined by means of the equations in Table 1 to compose the five possible outcome groups. This is illustrated in a fictive cohort of 1000 women receiving adjuvant RT for their stage 1 intermediate-risk endometrial carcinoma (Figure 1). This shows that from a putative cohort of 1000 patients, adjuvant RT will prevent recurrences in 60 patients (groups 1 and 2), a majority of 908 patients will neither benefit nor suffer severe radiation-induced harm (group 3), but 28 patients will suffer severe complications due to adjuvant RT (group 4) and an expected four patients will die (group 5).

## Discussion

In this study, a scheme is presented to balance the benefit and harm of adjuvant therapy *vs* no adjuvant therapy. To exemplify this scheme, we utilised the data on adjuvant pelvic external beam RT for intermediate-risk stage I endometrial carcinoma patients. The scheme readily provides an insight into the proportion of patients who will benefit from adjuvant therapy (some at the cost of complications) *versus* those who neither benefit nor contract complications, and those who do not benefit from adjuvant therapy but do contract severe complications, or even die.

It may be hard to imagine that providing an adjuvant therapy does not necessarily lead to the intended effect and can even harm our patients. However, as complications might harm patients who do not have any benefit from adjuvant therapy, we advocate the evidence as presented in this scheme to be communicated to them. This will enable patients to determine their preference regarding adjuvant therapy based on comprehensive information, which ultimately facilitates shared decision making (Charles *et al*, 1997; van Tol-Geerdink *et al*, 2008).

In our example of intermediate-risk stage I endometrial carcinoma patients, here is what one could communicate about adjuvant RT based on the insight provided by the completed scheme. The patient should first be informed about the risk of developing a local recurrence, that is about 10% according to our included studies. Providing adjuvant RT will not improve the patient's chances of survival, but does decrease the risk of developing a local recurrence. Along with communicating the probability of developing a local recurrence, one should try and explain to the patient what it is like to experience this. Subsequently, our scheme may be used by clinicians to explain that in a group of 1000 patients like them, if they would undergo postoperative RT, the majority (908) will neither benefit from RT in terms of prevented local recurrences, nor suffer from severe complications. The physical and mental discomfort of a local recurrence will be prevented in 60 patients, whereas serious, sometimes even life-threatening, complications from RT will occur in 28 patients, and an expected four patients will die. An explanation of what it would be for the patient to experience severe complications is needed, and it should be stressed that severe complications are included in the scheme (because these complications could cause them to reconsider having RT or not), but moderate and mild complications are not.

By means of this scheme, benefit and harm of two adjuvant therapies, or an adjuvant therapy *versus* no adjuvant treatment, can be balanced. Although this provides a convenient and orderly overview, the scheme is of less value in case more adjuvant therapies are available. For example, for stage I intermediate-risk endometrial cancer patients, vaginal intracavitary brachytherapy is currently hypothesised to be superior to external RT in terms of morbidity and mortality due to its local rather than regional effect (http://www.trialregister.nl/trialreg/admin/rctview.asp?TC=332). These two adjuvant therapies could be compared in a scheme as presented in this paper; however, the option of providing no adjuvant treatment cannot be incorporated in the same scheme. This leads to three separate schemes comparing: (1) external beam RT *vs* no adjuvant therapy, (2) brachytherapy *vs* no adjuvant therapy and (3) external beam RT *vs* brachytherapy, which is far too complicated to gain a view of the situation.

As the development of local recurrences is nowadays considered to be the primary end-point in studies of adjuvant RT, we chose local recurrence rate as the primary end-point in our scheme as well. However, we realise that this is an intermediate end point as it does not include subsequent benefit and harm of therapy after the development of a recurrence. Salvage therapy includes surgery, external beam RT and intracavitary RT, and is experienced as physically extremely uncomfortable and mentally very stressful, causing a substantial decrease in quality of life. Survival after relapse is significantly better in patients who have not received adjuvant RT than in those who already did (3-year survival 51 *vs* 19% (*P*=0.004)) (Creutzberg *et al*, 2003). However, this does not lead to a better overall survival in patients without adjuvant RT. In the scheme presented, it was impossible to incorporate the morbidity and mortality of treating a local recurrence by means of salvage therapy, although it is likely that the clinical bottom line would hardly change due to the great share of patients receiving neither benefit nor harm from adjuvant RT. When deciding about adjuvant RT, patients should therefore also be informed about the procedure of salvage therapy and the differences in survival after relapse.

The scheme as presented in this paper readily summarises the different possible treatment outcomes and can be of practical value for clinicians as well as for patients in (shared) decision making about adjuvant therapies. Moreover, it can be, and has already been in a few instances, generalised towards other interventions in other medical specialties, when pros and cons need to be weighed to make a treatment decision. Future research should reveal if clinicians as well as patients evaluate this scheme as a valuable tool to balance benefit and harm and whether it affects their final treatment decision.

## Figures and Tables

**Table 1:** Five groups of possible outcomes after adjuvant RT

**Group**	**Definition in this analysis**	**Equation**
1: Full benefit, no harm	Patients in whom RT prevented the development of a local recurrence and who do not suffer from morbidity due to RT	ARR × (1−ARI_morbidity_)
2: Benefit with harm	Patients in whom RT prevented the development of a local recurrence, but suffer from morbidity induced by RT	ARR × ARI_morbidity_
3: Neither benefit nor harm	Patients in whom RT did not prevent a local recurrence, but do not suffer from RT-related morbidity	(1−ARR) × (1−(ARI_morbidity_+ARI_mortality_))
4: No benefit but harm	Patients in whom RT did not prevent a local recurrence, and suffer from morbidity due to RT	(1−ARR) × ARI_morbidity_
5: Full harm	Patients who die as a result of RT	ARI_mortality_

ARI_morbidity_=absolute risk increase in radiation-induced morbidity; ARI_mortality_=absolute risk increase in radiation-induced mortality; ARR=absolute risk reduction in the development of local recurrences; RT=radiotherapy.

**Table 2:** Results of the included trials and overall risk differences

	***n***	**Local recurrences (%)**	**Severe morbidity (%)**	**Mortality (%)**
*PORTEC-1*				
Radiotherapy	354	11 (0.03)	6 (0.02)	1 (0.003)
Control	360	40 (0.11)	1 (0.003)	0 (0.00)
				
*GOG 99*				
Radiotherapy	190	3 (0.02)	9 (0.05)	2 (0.01)
Control	202	18 (0.09)	1 (0.005)	0 (0.00)
				
*ASTEC/EN.5*				
Radiotherapy	452	13 (0.03)	34 (0.08)	1 (0.002)
Control	453	29 (0.06)	15 (0.03)	0 (0.00)
				
***Overall***				
**Radiotherapy**	**996**	**27 (0.03)**	**49 (0.05)**	**4 (0.004)**
**Control**	**1015**	**87 (0.09)**	**17 (0.02)**	**0 (0.000)**
				
**Risk difference (95% CI)**	**2011**	**ARR: 0.06 (0.04–0.08)**	**ARI_morbidity_: 0.03 (0.02–0.05)**	**ARI_mortality_: 0.004 (0.000–0.008)**

ARI_morbidity_=absolute risk increase in radiation-induced morbidity; ARI_mortality_=absolute risk increase in radiation-induced mortality; ARR=absolute risk reduction in the development of local recurrences. 95% CI=95% confidence interval. Bold represent the pooled estimates of the three studies described above (PORTEC-1, GOG99 and ASTEC/EN.5).
